# Potential roles of tumor microenvironment in gefitinib-resistant non-small cell lung cancer: A narrative review

**DOI:** 10.1097/MD.0000000000035086

**Published:** 2023-10-06

**Authors:** Mu-Tong Chen, Bai-Zhi Li, En-Pu Zhang, Qing Zheng

**Affiliations:** a Department of Urology, The Third Affiliated Hospital of Shenzhen University (Luohu Hospital Group), Shenzhen, China; b Shantou University Medical College, Shantou, China.

**Keywords:** drug resistance, gefitinib, non-small cell lung cancer, targeted therapy, tumor microenvironment

## Abstract

During the course of treating non-small cell lung cancer (NSCLC) with epithelial growth factor receptor (EGFR) mutant, gefitinib resistance (GR) is unavoidable. As the environment for tumor cells to grow and survive, tumor microenvironment (TME) can significantly affect therapeutic response and clinical outcomes, offering new opportunities for addressing GR. Dynamic changes within the TME were identified during the treatment of gefitinib, suggesting the close relationship between TME and GR. Various dynamic processes like angiogenesis, hypoxia-pathway activation, and immune evasion can be blocked so as to synergistically enhance the therapeutic effects of gefitinib or reverse GR. Besides, cellular components like macrophages can be reprogrammed for the same purpose. In this review, we summarized recently proposed therapeutic targets to provide an overview of the potential roles of TME in treating gefitinib-resistant NSCLC, and discussed the difficulty of applying these targets in cancer treatment.

## 1. Introduction

One of the most prevalent malignant tumors in the world is lung cancer (LC), which ranks first by world cancer incidence and caused 17.6 million deaths in 2018.^[1]^ About 85% of LC subtypes are non-small cell lung cancer (NSCLC) which includes adenocarcinoma, squamous cell carcinoma, and large cell carcinoma. Although most early-stage NSCLC patients can be clinically cured through surgical excision, advanced-stage patients commonly require comprehensive management, like chemotherapy and radiotherapy.^[2]^ Due to the advances in molecular diagnosis, specific genetic changes such as epithelial growth factor receptor (EGFR) mutations, anaplastic lymphoma kinase (ALK) rearrangements, and Kirsten rat sarcoma viral oncogene homolog (KRAS) G12C mutations can be detected in patients’ blood or resected samples, which determines administration of corresponding molecular therapy.^[3]^ EGFR mutation had the highest detection rate among the above 3, especially in Asian patients (40%–55%). The positive result for typical EGFR mutations, including L858R and exon 19 deletions, indicates a sensitivity to gefitinib.^[4–8]^ As one of the first-generation EGFR tyrosine kinase inhibitors (EGFR-TKIs), gefitinib exhibits antitumor activity through competitively binding to the ATP-binding pocket of the EGFR domain and subsequent blockage of downstream proliferative signaling.^[9]^ Although the efficacy and safety of gefitinib are well-evaluated during long-term clinical practice, acquired gefitinib resistance (GR)—which denotes a systemic progression of the disease—frequently occurs about 9 to 10 months following gefitinib treatment.^[10–13]^ EGFR T790M mutation, which means an “off-target phenomenon” caused by steric hindrance effect in ATP pocket due to methionine replacement of threonine at position 790 on exon 20 of the EGFR gene, constitutes the main mechanism of NSCLC acquired GR.^[14]^ Although third-generation EGFR-TKI Osimertinib is developed and recommended for overcoming acquired GR, it comes with enormous consumption of time and financial resources.^[8,14]^ Furthermore, acquired resistance still occurs after about 11 months of Osimertinib treatment.^[14–16]^ Besides T790M mutation, other mechanisms for acquired GR include small cell histology transformation (10%), human EGFR 2 amplification (12%) mesenchymal–epithelial transition factor (MET) amplification (5%), and other undefined mechanisms (20%), to which general solutions are still lacking.^[14]^

Recently, the complexity of the tumor microenvironment (TME) and its regulatory role in cancer biological behaviors have been well-studied. TME is described as a complex consisting of various non-tumor cells and non-cellular components, which actively interact with tumor cells during oncogenesis, tumor progression, and the development of drug resistance.^[17,18]^ In addition, the physical and chemical properties like hypoxia and acidity also play roles as TME components. Furthermore, some TME components have been proven to mediate acquired GR in NSCLC through Ligand-receptor interaction and activation of downstream signaling, which can be targeted with drugs that have been clinically validated for safety and subsequently reshaped TME into a state favorable for therapeutic response.^[19]^ In addition, NSCLC TME showed temporal heterogeneity during gefitinib administration: a narrow time window characterized by increased CD8 + T cells and decreased Treg cells in the early stage, which is potentially favorable for the combination with Programmed cell death receptor-1/ programmed cell death ligand-1 (PD1/PD-L1) blockade. However, this time window was quickly missed and replaced by a period characterized by high infiltration of myeloid-derived suppressor cells, which contribute to an immunosuppressive environment.^[20]^ Although the dynamic changes of TME in the process of developing resistance have not been studied, this property should be emphasized when designing therapeutic strategies. Therefore, the identification of novel targets and the development of novel combinations and sequential strategies to remodel TME is of great potential.

**Figure 1.**
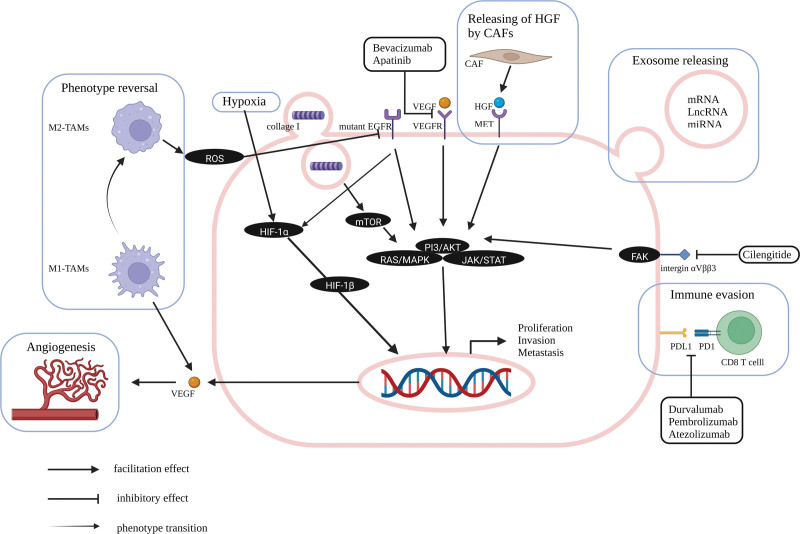
Schematic illustration of the regulatory mechanism of EGFR mutation and TME components on NSCLC cells (Created with BioRender.com). EGFR mutation can activate downstream signaling, which facilitates the proliferation, invasion, and metastasis of NSCLC cells. VEGF/VEGFR pathways share common downstream signaling with EGFR and can be blocked with Bevacizumab and Apatinib. The HIF-1α pathway induced by hypoxia is also regulated by the mutant EGFR pathway. The phenotype of macrophages can be artificially converted from M1 into M2 and then increase the release of ROS for resolving T790M EGFR protein. PDL1 is up-regulated in NSCLC cells harboring EGFR mutation for facilitating immune evasion. Recently clinical trials suggest that ICIs, including Durvalumab, Pembrolizumab, and Atezolizumab, are more effective in treating EGFR mutant patients when combined with chemo or antiangiogenic rather than combined with gefitinib. HGF, which can be secreted by CAFs, is able to induce GR through binding with MET protein. NSCLC cells can secrete various exosomes containing miRNA, lncRNA, and mRNA to affect GR through different mechanisms. Collagen type I can be intake by NSCLC cells and induce GR through the activation of mTOR and downstream EGFR signaling. The up-regulation of integrin αVβ3 can confer GR through FAK/AKT signaling. EGFR = epidermal growth factor receptor, CAFs = cancer-associated fibroblasts, FAK = focal adhesion kinase, GR = gefitinib resistance, HIF-1α = hypoxia-inducible factor-1α, HGF = hepatocyte growth factor, MET = epithelial-mesenchymal transition, mTOR = mammalian target of rapamycin, NSCLC = non-small-cell lung cancer, PDL1 = programmed cell death 1, ROS = reactive oxygen species, TAMs = tumor-associated macrophages, TME = tumor microenvironment, VEGFR = vascular endothelial growth factor receptor, .

In this article, we reviewed recent studies related to potentiating the therapeutic effect of gefitinib as well as reversing GR and summarized the therapeutic targets proposed. In addition, an overall schematic diagram and a summarized table were provided for a better understanding of their mechanisms (Fig. 1 and Table 1).

**Table 1:** The summary of TME-related targets in EGFR mutant NSCLC.

TME components		Targets	Description	Phase	Ref
Vasculature and hypoxia		VEGF/VEGFR	A typical regulator of angiogenesis, which share common downstream signaling with EGFR pathway in cancer cells.	Clinical	^[24,30,32,33]^
		HIF-1α pathway	A typical pathway activated in hypoxic status in cancer cells, which can be activated by EGFR pathway	Preclnical	^[26,27]^
Immune system		TAMs	TAMs can be reprogrammed from M2 phenotype to M1 phenotype, which promote the degradation of EGFR.	Preclnical	^[44,45]^
		PD-1/PD-L1	A typical regulator of immune evasion, its theraputic effect when being targeting is associted with the status of immune-microenvironment	Clinical	^[58–65]^
CAFs		HGF	A kind of growth factor that secrected by CAFs, which can bind to c-myc protein on the surface of CAFs	Preclnical	^[71,72,74]^
Exosome	mRNA	GK5	Inducing GR through activating the SERBP1/SCD1 pathway	Preclnical	^[86]^
	LncRNA	H19	Inducing GR	Preclnical	^[88]^
		UCA1	Inducing GR through sponging miR-143 and subsequently up-regulated FOSL2 expression	Preclnical	^[87]^
	miRNA	miR-7	reversing GR for H1975 cells	Preclnical	^[84]^
		miR-214	Inducing GR	Preclnical	^[85]^
Extracellular matrix		Collagen type I	Inducing GR through the activation of EGFR downstream signaling	Preclnical	^[91,92]^
		Integrin αVβ3	inducing GR through FAK signaling	Preclnical	^[96,97]^

CAFs = cancer-associated fibroblasts, EGFR = epidermal growth factor receptor, FAK = focal adhesion kinase, GR = gefitinib resistance, HGF = hepatocyte growth factor, HIF-1α, hypoxia inducible factor-1α, NSCLC = non-small-cell lung cancer, PDL1, programmed cell death 1, TAMs = tumor associated macrophages, TME = tumor microenvironment, VEGFR = vascular endothelial growth factor receptor.

## 2. Methods

We performed a comprehensive PubMed search between January and February 2022 to identify available studies regarding the role of TME in gefitinib-resistant NSCLC. If available, we used keywords and the corresponding Mesh terms: Non-small cell lung cancer, EGFR, Tumor micro-environment, GR, Vasculature, Angiogenesis, Hypoxia, Immune, Fibroblast, Exosome, and Extracellular matrix. All original articles and reviews investigating the role of TME in gefitinib-resistant NSCLC were considered to obtain a complete and in-depth perspective on this topic. Non-English full texts were excluded. The timeframe included in the obtained articles was until 2022. Two independent reviewers (Chen and Li) appraised and included the studies based on the title, abstract and full text of the original papers. If a discrepancy of opinion occurs between 2 reviewers, the third reviewer (Zheng) will decide on the literature inclusion. In addition, relevant original articles cited by searched review will be included. The detailed search process is presented in Table 2 and Supplemental Table 1 (see Supplemental Table 1, Supplemental Content, http://links.lww.com/MD/J789 which illustrates the detailed search process). This article contains no studies with human participants or animals performed by any of the authors. Therefore, no ethical approval or consent is required.

**Table 2:** The search strategy summary.

Items	Specification
Date of search	From June 1, 2022, to July 26, 2022
Databases and other sources searched	Pubmed (https://pubmed.ncbi.nlm.nih.gov/)
Search terms used	Following keywords and corresponding Mesh terms, if available, were used: Non-small cell lung cancer, Epidermal growth factor receptor, Tumor micro-environment, Gefitinib resistance, Vasculature, Angiogenesis, Hypoxia, Immune, Fibroblast, Exosome, and Extracellular matrix. The detailed search strategy was demonstrated in the supplement table.
Timeframe	Until 2022.
Inclusion and exclusion criteria (study type, language restrictions, etc)	Both original articles and reviews were included. Only full English texts were included. Non-English full texts were excluded.
Selection process	Two independent reviewers (M.C. and B.L.) appraised and included the studies based on the title, abstract, and full text of the original papers. Decisions on the discrepancy of opinions were made by Q.Z.
Any additional considerations, if applicable	An additional search for content supplements was conducted, if needed. Suitable original articles cited by relative review will be included.

## 3. Vasculature and hypoxia

### 3.1. Angiogenesis and hypoxia

Angiogenesis is considered one of the tumor hallmarks for its role in forming a persistent nutrition supply, and it regulated by multiply growth factors, in which the vascular endothelial growth factor (VEGF) is considered a typical mediator in this process.^[17,21]^ In hypoxic status caused by rapid growth and proliferation of tumor cells, oxygen-regulated hypoxia-inducible factor 1α (HIF-1α) can avoid degradation and then dimerize with HIF1β, subsequently activating the transcription of downstream genes, including VEGF.^[22,23]^ VEGF is majorly secreted by tumor cells, subsequently binding to vascular endothelial growth factor receptor (VEGFR) in endothelial cells, contributing to the generation of vasculature in TME. In NSCLC cells, the VEGF/VEGFR pathway shares common downstream signaling with EGFR, indicating its additional role in supporting tumor growth when EGFR is blocked. In NSCLC cells harboring EGFR mutation (including T790M), VEGFR/VEGFR interaction activates EGFR downstream signaling pathways, such as RAS/MAPK and PI3K/AKT, subsequently activating gene transcription involving in proliferation, invasion, and angiogenesis.^[24,25]^ In addition, the HIF-1α pathway induced by hypoxia is regulated by mutant EGFR, and a positive feedback loop existing between HIF-1α and c-Jun protein is also proven to promote resistance to gefitinib.^[26,27]^ Therefore, targeting VEGF/VEGFR and HIF-1α in the presence of gefitinib holds the potential to overcome acquired GR in EGFR mutant NSCLC.

Bevacizumab, an intravenously administered recombinant human immunoglobulin G1 monoclonal antibody, can bind to VEGF-A and prevent it from binding to VEGFR-2, hence blocking any following biological effects.^[28]^ The effects of the combination of gefitinib and bevacizumab have not yet been fully understood, despite the fact that the reinforced effect of bevacizumab plus the first-generation EGFR-TKI erlotinib has been demonstrated and was even recommended by the NCCN guideline as the first treatment for NSCLC harboring the typical EGFR mutation.^[8,29]^ Patients harboring typical EGFR mutation who were in stage IIIB/IV or had recurrence were enrolled in a randomized phase II trial by CHIYOE KITAGAWA et al, and the median progression-free survival (PFS) for gefitinib alone versus gefitinib plus bevacizumab is 15.1 (80%CI = 6.2–23.5) months versus 5.4 (80%CI = 5.0–13.9) months, potentially indicating the unavailability of this combination.^[30]^ However, the study sample size (16 patients) falls short of the number anticipated in advance, and the early termination also renders the finding implausible. Therefore, the combination of gefitinib with bevacizumab still requires further explorations in NSCLC patients harboring GR.

Apatinib is a multitarget TKI targeting VEGFR1, VEGFR2, c-RET, c-KIT, and c-SRC, which can be administered orally.^[31]^ In a randomized phase 3 study conducted by Hongyun Zhao et al, harboring typical EGFR mutation, treatment-naïve, and advanced stage, NSCLC patients were enrolled, and median PFS was improved with Apatinib plus gefitinib as compared with placebo plus gefitinib (13.7 months vs 10.2 months, hazard ratio = 0.71, *P* = .0189).^[32]^ Although a minor increase in the incident rate of adverse events without affecting life quality was observed and a promoted overall survival still to be confirmed, the prospective benefits of this combination in patients with T790M remain worth further investigation.^[33]^ In another study conducted by Fang Li et al, this combination shows a reinforced effect on inhibiting EGFR and VEGFR2 phosphorylation, tumor growth, and angiogenesis in NSCLC harboring T790M. In a retrospective analysis of 16 patients who primarily acquired typical EGFR mutation and developed resistance after first-line treatment of different EGFR-TKIs, the second-line treatment with the combination of EGFR-TKIs and Apatinib confers a median PFS of 4.6 months (IQR, 4.08–10.97 months) and 100% of disease control rate.^[33]^ Although the data used as clinical verification of this combination is retrospective and other EGFR-TKIs are involved, this study still points out the potential of Apatinib in overcoming acquired GR when combined with gefitinib. In addition, ChiCTR1800019185 is a single-arm clinical trial that evaluates the PFS of combining Apatinib with EGFR TKI in NSCLC with slow progression following EGFR-TKI first-line therapy.^[34]^

### 3.2. Vascular contraction

In addition, Vascular contraction also confers the development of GR in NSCLC as its role in reducing drug penetration. Endothelin is known as a potent vasoconstrictor, while endothelin-1(EDN1) is one of the endothelin family and is related to poor prognosis in NSCLC patients.^[35,36]^ Although the binding of EDN1 to its receptor EDNRA additionally leads to the activation of MAPK-ERK signaling, which is a tumor-promoting pathway, most strategies targeting EDN1 based on this premise end up with unfavorable results.^[37–41]^ In a study accomplished by Inés Pulido et al, a subclone harboring exon 19 deletion and EMT phenotype holds an up-regulation of EDN1 was identified after exposure to erlotinib (another first-generation EGFR-TKI).^[42]^ This subclone did not show a growth advantage in vitro and vivo but was proved to mediate GR through vasoconstriction and reduced blood supply without changing the vascular density, and this effect can be reversed by injection of EDNR antagonist bosentan.^[42]^ Although the result of this study cannot be easily applied to clinical practice, it points out that TME components can mediate GR independently of EGFR mutation.

## 4. Immune system

### 4.1. Tumor-associated macrophages

All the immune cells can be discovered in TME, which are actively interacting with cancer cells as well as intervening in therapeutic response through secreting soluble factors or direct contact^[17]^ as one of the major stromal cells within TME, tumor-associated macrophages (TAMs) are considered to be an important mediator of GR. TAMs can be primarily divided into 2 categories based on their phenotype: M1 as tumor-suppressor and M2 as tumor-promotor.^[43]^ In NSCLC, the reversal of the M1 phenotype to M2 is proved to be effective in overcoming T790M GR.^[44,45]^ Methionine sulfoxide reductase A (MsrA) is a protector of the T790M EGFR protein. Reactive oxygen species (ROS) are produced at higher levels as a result of the phenotypic switch from M2 to M1, which causes MsrA to be down-regulated and subsequently accelerates the degradation of EGFR.^[44,45]^ Huige Peng et al developed a trastuzumab-modified, mannosylated liposome carrying gefitinib and vorinostat, in which trastuzumab is for targeting her2-positive NSCLC cells, and vorinostat is for TAMs phenotype reversal.^[44]^ Weimin Yin et al developed a PD-L1-modified liposome carrying gefitinib and simvastatin, in which simvastatin is for targeting TAMs.^[45]^ The results showed that the reversal of TAMs phenotype leads to not only up-regulation of ROS production but also inhibition of angiogenesis and increased release of pro-inflammatory factors like TGF-β. Both 2 nano-drugs show superior tumor-killing effects and favorable safety in mouse models harboring T790M NSCLC. The results above demonstrated the potential of overcoming GR through manipulating TAMs phenotype and remodeling TME in NSCLC.

In addition, TAMs can confer GR to EGFR-TKIs sensitive NSCLC. Epiregulin is one of the EGFR ligands, and in NSCLC patients using erlotinib, its up-regulation is linked to an unfavorable prognosis.^[46]^ A study conducted by Shiqi induced THP-1 cells and peripheral blood monocytes into macrophages and polarized into M1 as well as M2 phenotype.^[46]^ Subsequent experiments proved that both M1 and M2 macrophages confer GR to gefitinib-sensitive NSCLC cells through paracrine secretion of Epiregulin and activation of EGFR/ErbB2 dimerization as well as downstream signaling, while the up-regulation of Epiregulin in NSCLC cells cannot mediate this process.^[46]^ This study suggests the importance of excavating explanations from TME components when discovering the association between unfavorable prognosis and up-regulated genes in tumor tissue.

### 4.2. Immune evasion

Immune escape is considered one of the cancer hallmarks.^[47]^ PD1 is a typical immune checkpoint mediating immune escape for cancer cells because its binding with PD-L1 from tumor cells results in the inhibition of CD8 T-cell tumor-killing activity.^[48]^ However, the binding between PD-1 and PD-L1 also shields normal cells from being attacked by the immune system, which potentially explains the toxicity from PD-1/PD-L1 target therapy.^[49]^

Preclinical research on NSCLC demonstrated a positive correlation between EGFR mutation and the up-regulation of PD-L1 expression as well as a PD-L1 down-regulation by administration of EGFR-TKIs.^[50–52]^ Although this correlation suggests cooperativity for combined PD1/PD-L1 target therapy with gefitinib, no cooperative therapeutic effects could be observed from EGFR mutant NSCLC cell lines nor patients regardless of PD-L1 status.^[50,53–55]^ Although the subtyping of NSCLC transcriptome data suggests that most NSCLC subtypes are associated with enriched immune components as well as various immune checkpoints, lymphocyte-depleted characteristics of EGFR-driven NSCLC was identified in another largest clustering of pan-cancer multi-omics data, which suggests the natural insensitivity to immunotherapy.^[56,57]^ In a phase 1 trial conducted by Benjamin C. Creelan et al, thirty TKI-naïve NSCLC patients with typical EGFR mutation were enrolled and administrated with the combination of gefitinib and PD-L1 monoclonal antibody durvalumab for the evaluation of the effectiveness and safety.^[54]^ The results showed that this combination brought no significant improvement in PFS (10.1 months, 95% CI: 5.5–15.2) as well as objective response rates (ORRs, 63.3%) but increased toxicity like elevated aspartate aminotransferase (60%) and diarrhea (78%) when compared to historical data of gefitinib monotherapy.^[54]^ In another phase 1/2 trial conducted by James Chih-Hsin Yang et al, the administration of gefitinib plus PD-1 monoclonal antibody pembrolizumab was given to a similar population(n = 7), and the results also demonstrated that the combination of PD-1/PD-L1 target therapy with gefitinib only increases toxicity instead of efficacy: 1.4 months (95% CI: 0.2–13.0) for PFS as well as 14.3% for ORRs and 71.4% for elevated aspartate aminotransferase incident rate.^[53]^ A plausible explanation for these results is that the administration of EGFR-TKIs leads to an immune-suppressive environment characterized by significant myeloid-derived suppressor cell accumulation.^[20,50]^ In addition, the down-regulation of PD-L1 caused by EGFR-TKIs leads to an “off-target” phenomenon for PD-1/PD-L1 blockage, which partially explains the ineffectiveness and increased toxicity.^[49,50]^ Collectively, the combination of gefitinib with PD-1/PDL-1 target therapy is inappropriate as first-line treatment for NSCLC patients harboring EGFR mutation due to its disability of activating tumor-killing by the immune system.

Despite disappointing results with PD-1/PD-L1 target therapy as a single agent and in combination with gefitinib in EGFR-mutant NSCLC patients, PD-1/PD-L1 target therapy in combination with chemo or antiangiogenic agent provides some hope for the treatment of patients developing GR.^[58–65]^ In a phase 2 trial PROLUNG conducted by Oscar Arrieta et al, 25 patients with EGFR mutation were included after platinum-based chemotherapy failure, and the results revealed that the combination of pembrolizumab plus chemo-agent docetaxel exhibits an improved PFS compared with docetaxel alone without significantly increased toxicity (6.8 months, 95% CI, 6.2-not reached; vs 3.5 months, 95% CI, 2.3–6.2).^[58]^ Another retrospective study conducted by Sangtian Liu et al concluded that patients with shorter PFS after EGFR-TKIs treatment are preferable to receive a second-line treatment that combines PD-1/PD-L1 target therapy plus chemotherapy (median PFS, 15.1 vs 3.8 months), and these patients have a higher infiltration of CD8 T cells and M2-TAMs.^[65]^ In phase 3 trial IMpower150 conducted by Martin Reck et al, 124 patients harboring EGFR mutation were included and distributed to 3 groups: for groups ABCP, ACP, and BCP, respectively, atezolizumab (anti-PD-L1) plus bevacizumab (anti-VEGF) plus carboplatin plus paclitaxel, atezolizumab plus carboplatin plus paclitaxel and bevacizumab plus carboplatin plus paclitaxel.^[59]^ The results show that the group ABCP holds a longer PFS (10.9 months, 95%CI 7.9–15.2) and similar incident rate of toxic events when compared with group BCP (6.9 months, 95%CI 5.7–8.5) or ACP (6.9 months 95%CI 5.7–8.2).^[59]^ Based on promising results from IMpower150, T.C. Lam and his colleagues conducted a phase II trial to assess the effectiveness of this combination on 40 metastatic patients who developed resistance to at least one EGFR-TKI.^[66]^ Paclitaxel in the previous combination was replaced by pemetrexed to improve tolerance, and the results show 9.4 months of PFS (95% CI:7.6–12.1), which is close to historical data of Osimertinib on treating T790M patients.^[14–16,66]^ Mechanisms behind these results are potentially related to a “re-inflamed” immune-microenvironment. Firstly, the chemo-agent leads to enhanced release of tumor-associated antigens through inducing immunogenic death of tumor cells and subsequently increases the recruitment of immune cells.^[67,68]^ Secondly, the addition of an anti-VEGF agent leads to a normalization of vasculature within TME as well as promoting leukocyte adhesion through blocking VEGF signaling on immune cells, both of which lead to an increased abundance of immune infiltration.^[69]^ Although the specific mechanism behind this remains to be explored, re-activating immune-suppressive TME through the addition of agents targeting other TME components is of great potential.

## 5. Cancer-associated fibroblasts

### 5.1. Hepatocyte growth factor (HGF) is a key factor from cancer-associated fibroblasts (CAFs) in mediating GR

Besides TAMs, CAFs are another main component of stromal cells, which were proven to mediate GR through direct contact with tumor cells and the secretion of soluble factors.^[70–74]^ MET transmembrane protein is encoded by the MET gene, and its phosphorylation leads to the activation of downstream signaling, including MAPK, PI3K, STAT, etc, which is shared with EGFR signaling.^[75–77]^ MET factor amplification, which can be detected in about 5% of patients developing resistance to first-generation EGFR-TKIs, is another important mechanism for GR.^[78,79]^ Similarly, the binding of HGF to MET protein can induce activation of the same signaling, which can then bypass the blockage and result in GR.^[73,80]^ In several studies, CAFs were proved to be a crucial source of HGF rather than tumor cells as well as endothelial cells, therefore becoming a facilitator for GR.^[71,72,74]^ In an initial CAFs study conducted by Wei Wang et al, patient-derived CAFs from 5 lung cancer patients, 1 line of human lung embryonic fibroblasts, and NSCLC cell lines with typical EGFR-mutation were used for the following experiments.^[72]^ They discovered that HGF could mediate GR through binding to MET protein as well as activating downstream PI3K/Akt signaling, and the blockage of HGF when co-culturing tumor cells with CAFs causes the removal of previous effects.^[72]^ In another study conducted by Yanmei Yi et al, tumor specimens with EGFR 19del were used for the extraction of CAFs.^[71]^ Similar results to Wei Wang et al‘s study were observed: the co-existence of gefitinib-sensitive NSCLC cells and CAFs leads to the improvement of cell viability, promotion of MET, as well as resistance to gefitinib.^[71]^ Furthermore, they discovered that insulin-like growth factor 1(IGF-1) is another paracrine factor from CAFs that may play an auxiliary role for HGF, and Annexin A2 (ANXA2) is a required factor located downstream when IGF-1, as well as HGF, mediate GR.^[71]^ However, the specific mechanism by which ANXA2 regulates GR in NSCLC remains unknown. The results of the study by Erika Suzuki et al show that not all patient-derived CAFs can mediate GR.^[74]^ In Erika Suzuki study, CAFs, as well as paired normal fibroblasts (PNFs), were collected from 18 patients, and the results demonstrated that not all CAFs could promote GR compared to PNFs.^[74]^ However, consistent with previous results, HGF serves as a marker for resistance-promoting CAFs, associated with the frequency of local invasion in NSCLC patients.^[74]^ Overall, the results above indicate that CAFs mediate GR through secreting HGF as well as other auxiliary factors, which potentially explain some undefined resistance mechanisms.

### 5.2. Heterogeneity of CAFs

From resected materials, patient-specific CAFs and their great heterogeneity in mediating GR were noted. Tatsuya Yoshida et al discovered that when CAFs with up-regulation of podoplanin were co-cultured with gefitinib-sensitive NSCLC cells (harboring typical EGFR mutations), there was an increase in Akt and ERK phosphorylation, and the cells became resistant to gefitinib compared to another co-cultured medium containing CAFs with mutant-podoplanin.^[70]^ In addition, this resistance was erased when co-cultured the tumor cells and CAFs in separate chambers, indicating that this resistance was achieved through direct contact.^[70]^ In a retrospective analysis of clinical data, podoplanin-CAFs in resected specimens were found to correlate with poorer response rates (53% vs 83%; *P* < .01) and shorter PFS (9.6 vs 15.6 months; HR, 1.763; 95% CI, 1.146–2.713; *P* = .0099) in patients previously received EGFR-TKIs as first-line treatment.^[70]^ However, in another study conducted by Masayuki Ishibashi et al, the opposite effect was identified from another subgroup of CAFs featured by up-regulation of CD200.^[81]^ This subgroup of CAFs enhanced the gefitinib sensitivity of NSCLC cells harboring typical EGFR mutation in co-cultured conditions, and their appearance in resected specimens was correlated with longer PFS for gefitinib treatment.^[81]^ Although the precise mechanisms by which CD200 and podoplanin affect GR as functional proteins as well as factors that cause heterogeneous phenotypes of CAFs, remain to be elucidated, more CAFs subgroups will be identified using newly-advanced single-cell RNA sequencing and will become potential predictors for EGFR-TKI response as well as therapeutic targets.^[82]^

## 6. Exosome

Exosomes are defined as membranous vesicles with a diameter ranging from 20–200nm, in which message RNA (mRNA), long-noncoding RNA (lncRNA) as well as microRNA (miRNA) are contained. Several studies have proved exosomes from GR cell lines are capable of transferring GR phenotype to sensitive cells through conferring miRNA. In the study conducted by Xiaozhen Liu et al, exosomes from NSCLC cells H1975 harboring T790M mutation were isolated and fed to gefitinib-sensitive PC9 cells.^[83]^ Results showed that exosomes successfully conferred GR to PC9 cells through activating PI3K/AKT signaling, and miR-522-3p was potentially responsible for this process, but further exploration of the detailed mechanism was skipped.^[83]^ In another study conducted by Rui Chen et al, the expression level of several miRNAs related to GR in the literature review, including miR-7, miR-19b, miR-17-3p, miR-34c, miR-25, and miR-181a were tested with qRT-PCR, and miR-7 was found to be the highest expressed miRNA in PC9 cells compared with H1975.^[84]^ A similar screening method was performed on identifying responsible signaling pathways, and the relationship between the Hippo pathway as well as YAP protein and miR-7 was confirmed by site prediction by an online website and luciferase reporter experiment.^[84]^ Subsequent experiments proved that overexpression or knockdown of miR-7 in somatic cells also leads to synchronized changes in exosomes, and exosomes containing miR-7 from PC9 cells were proved to reverse GR for H1975 cells.^[84]^ These 2 studies suggested a potential balancing of exosome-crosstalk between T790 M-resistant cells and gefitinib-sensitive cells. Besides GR induced by T790M, miR-214 was also shown to be a potential GR mediator in exosomes isolated from PC9 cells that induced resistance, which is a mechanism distinct from T790M-induced resistance.^[85]^

Besides miRNA, mRNA, and lncRNA have also been identified as GR mediators in preclinical studies. In Jian Zhou study, the exosomal mRNA level of Glycerol kinase 5 (GK5) in EGFR-TKIs resistant patients’ plasma was up-regulated compared with nonresistant patients.^[86]^ Although subsequent experiments confirmed that CK5 induced GR through activating the SERBP1/SCD1 pathway, several flaws like the opaque definition of EGFR-TKIs resistance patients, unknown EGFR mutation status for clinical samples, and no experiment to confirm exosome intake still exist.^[86]^ Further exploration of metabolic alteration led by the CK5 exosome is also required. Two studies explored the role of exosomal LncRNA in mediating GR, and H19, as well as UCA1, were identified to be of importance. In Xiliu Chen study, lncRNA UCA1 in exosome from induced-resistant PC9, as well as HCC827(another cell line harboring typical EGFR mutation), was proved to mediate GR through a competing endogenous RNA mechanism, which involved the sponge of UCA1 on miR-143 and subsequently up-regulate FOSL2 expression.^[87]^ Similarly, H19 from induce-resistant cell lines was proved to significantly affect GR, and Yi Lei and his colleagues also explored the detailed mechanism of the H19 packaging process.^[88]^ Although both studies above discovered novel exosomal targets, it is unclear whether these targets can be applied to NSCLC harboring T790M due to the method by which generating GR cell lines.

Undoubtedly, more nucleic acid and other components contained in exosomes that can significantly affect GR will be discovered, but the problems of how to translate these targets into clinical practice need to be considered.

## 7. Extracellular matrix (ECM)

ECM is believed to be the key of cancer management due to its roles in forming tissue structure and providing biophysical and biochemical cues for the enhancement of cancer hallmarks.^[89]^ ECM consists of collagen, fibronectin, mucins, vitronectin, tenascin-C, etc, some of which have been proven to affect GR in recent studies.^[90]^

### 7.1. Collagen type I

As the main matrix protein in ECM, collagen type I (COL I) was found to be relevant to GR. Based on the hypothesis that cells internalize ECM components as one method of nutrient supply, Shota Yamazaki and his colleague discovered that the macropinocytosis of COLI by PC9 cells is a novel mechanism for GR, which is achieved through the activation of mammalian target of rapamycin signaling.^[91,92]^ Similar results were obtained from Yuanyuan Wang study.^[93]^ The addition of COLI into the medium promoted both the survival of HCC4006 as well as H1975 during the administration of EGFR-TKIs in a dose-dependent manner. Co-culturing gefitinib-sensitive cells with resistance one or fibroblasts also increased the resistance, and this effect can be partly reversed by collagen synthesis inhibitors. Collectively, the COLI is a potential target for synergistically enhancing the gefitinib effect. In addition, the effect of other ECM components on gefitinib-administrated cells, like fibronectin and mucin, is waiting to be evaluated.

### 7.2. Integrin αVβ3

The integrin family is the main transmembrane protein mediating cell adhesion to ECM, which can be dimerized by various types of α and β subunits. The correlation between the integrin family and cancer malignant phenotypes, like local invasion and distant metastasis, has been confirmed, but the diversity of integrin ligands and the heterogeneity of integrin dysregulation indicate the importance of targeting specific integrin subunits.^[94]^ In addition, the focal adhesion kinase (FAK) protein is a typical downstream molecule that is phosphorylated following integrin activation and exhibits excellent potential for combinational strategy.^[94,95]^ Recent studies have identified integrinαVβ3 as a promising target for reversing GR. In Caiyun Wang study, up-regulation of integrin β3 subunit was proved to confer GR, proliferation, anoikis-resistance, migration as well as invasion ability, and tumor stemness, which were through activation of FAK signaling.^[96]^ In line with Wang study, Yulong Fu and his colleagues found that the up-regulation of integrinαVβ3 also confers EGFR-TKIs resistance through FAK/AKT signaling, and the blockage of AKT signaling reversal of the resistance to gefitinib.^[97]^ Similarly, according to research by Fei He et al, the up-regulation of integrin V3 in NSCLC cells causes EGFR-TKI resistance, including GR via activating Galectin-3/KRAS/RalB/TBK1/NF-κB signaling.^[98]^ Autocrine factors from tumor cells, including osteopontin and TGF-β, were identified as contributors to up-regulated integrin β3 as well as αV, which also exhibited the potential to be targeted.^[96,97]^ However, upregulation of integrins β1, α5, and α2 rather than β3 was discovered in erlotinib resistance cell lines and refractory tumor samples previously treated with erlotinib as well as gefitinib, and the integrin β1/Src/Akt signaling pathway was identified as a mechanism of EGFR-TKI resistance, which indicates that the therapeutic value of integrin αVβ3 is GR-specific.^[99]^ Although the drug cilengitide, which targets integrin V3, provided no additional clinical benefit in clinical trials for the treatment of glioblastoma, it showed a synergetic effect with the administration of gefitinib on EGFR-wild type NSCLC cells, which indicated the tempting prospect for further exploration.^[100–102]^

## 8. Conclusion

GR remains a significant challenge in the treatment of EGFR mutant NSCLC. Undoubtedly, the TME not only provides explanations for part of undefined GR but also provides a new perspective for enhancing the therapeutic effects of gefitinib and reverse GR. However, our cognitive level on the NSCLC TME is far lower than tumor cells. Although some strategies targeting TME are proven to be effective in the preclinical phase, their clinical value still requires further validation. Novel drugs for these targeting are waiting to be developed and thoroughly evaluated. Furthermore, a multitargeting approach against these targets may offer a more efficient way to treat EGFR mutant NSCLC. In addition, TME can be reprogrammed to a more active state for therapeutic response, which is superior to blocking a single target. Developing rational multidrug strategies based on existing drugs, or exploring sequential strategies, may represent a potential solution to overcome GR and improve patient outcomes.

## Author contributions

**Data curation:** Mu-Tong Chen, Bai-Zhi Li.

**Formal analysis:** Mu-Tong Chen, Bai-Zhi Li.

**Project administration:** Mu-Tong Chen, Bai-Zhi Li.

**Writing – original draft:** Mu-Tong Chen, Bai-Zhi Li.

**Writing – review & editing:** En-Pu Zhang, Qing Zheng.

## Supplementary Material
