# Supplementary material for: Potential roles of tumor microenvironment in gefitinib-resistant non-small cell lung cancer: A narrative review
Source: Medicine (Baltimore). 2023 Oct 6;102(40):e35086. doi: 10.1097/MD.0000000000035086 (PMC10553124; doi:10.1097/MD.0000000000035086)
Supplement: Supplementary file 1 [file medi-102-e35086-s001.docx]

**Supplemental Table 1. Detailed search strategy demonstration.**

| **Concept** | **Keyword** | **Mesh term** |
| --- | --- | --- |
| Non-small cell lung cancer | Non-small cell lung cancer | Carcinoma, Non-Small-Cell Lung |
| Epidermal growth factor receptor | Epidermal growth factor receptor | ErbB Receptors |
| Tumor micro-environment | Tumor microenvironment | Tumor Microenvironment |
| Gefitinib resistance | Gefitinib resistance | **Not available** |
| Vasculature | Vasculature | **Not available** |
| Angiogenesis | Angiogenesis | **Not available** |
| Hypoxia | Hypoxia | Hypoxia |
| Immune | Immune | **Not available** |
| Fibroblast | Fibroblast | Fibroblasts |
| Exosome | Exosome | Exosomes |
| Extracellular matrix | Extracellular matrix | Extracellular Matrix |
| **Final combination of search terms** | | |
| (("Carcinoma, Non-Small-Cell Lung"[Mesh]) AND ("ErbB Receptors"[Mesh]) AND Gefitinib resistance) AND (fibroblast OR ("Fibroblasts"[Mesh]) OR Hypoxia OR ("Hypoxia"[Mesh]) OR Vasculature OR Angiogenesis OR Immune OR Exosome OR ("Exosomes"[Mesh]) OR Extracellular matrix OR ("Extracellular Matrix"[Mesh]) OR Tumor microenvironment OR ("Tumor Microenvironment"[Mesh])） | | |
